# Incidence of Hospital-Acquired Pneumonia, Bacteraemia and Urinary Tract Infections in Patients with Haematological Malignancies, 2004–2010: A Surveillance-Based Study

**DOI:** 10.1371/journal.pone.0058121

**Published:** 2013-03-05

**Authors:** Catherine Huoi, Philippe Vanhems, Marie-Christine Nicolle, Mauricette Michallet, Thomas Bénet

**Affiliations:** 1 Infection Control and Epidemiology Unit, Edouard Herriot Hospital, Hospices Civils de Lyon, Lyon, France; 2 Epidemiology and Public Health Group, Centre National de la Recherche Scientifique, Unité Mixte de Recherche 5558, University of Lyon 1, Lyon, France; 3 Haematology Department, Edouard Herriot Hospital, Hospices Civils de Lyon, Lyon, France; California Department of Public Health, United States of America

## Abstract

**Objective:**

This study charted incidence trends of hospital-acquired (HA) pneumonia, bacteraemia and urinary tract infections (UTI) in a haematology department.

**Methods:**

Prospective surveillance of hospital-acquired infections (HAI) was undertaken in a 42-bed haematology department of a university hospital. All patients hospitalized ≥48 hours between 1^st^ January 2004 and 31^st^ December 2010 were included. Definitions of HAI were based on a standardized protocol. The incidence was the number of events per 1000 patient-days at risk; only the first HAI was counted. Multivariate Poisson regression was fitted to assess temporal trends.

**Results:**

Among 3 355 patients (58 063 patient-days at risk) included, 1 055 (31%) had HAI. The incidence of HA pneumonia, HA bacteraemia and HA UTI was respectively 3.3, 12.0 and 2.9 per 1000 patient-days at risk. HA bacteraemia incidence increased by 11% (95% confidence interval: +6%, +15%, *P*<0.001) per year, independently of neutropenia, central venous catheterization (CVC) and haematological disease. The incidences of HA pneumonia and HA UTI were stable. The most frequently isolated pathogens were *Aspergillus spp*. (59.2%) for pneumonia, coagulase-negative *Staphylococcus* (44.2%) for bacteraemia and enterobacteria (60%) for UTI.

**Conclusion:**

The incidence of bacteraemia increased, indicating that factors other than CVC exposure, including chemotherapy with its impact on the immune system, could explain this trend. Further analytic studies are needed to explore the factors that could explain this trend.

## Introduction

Patients with haematological malignancies are at high risk of hospital-acquired infections (HAI) because the severity of their underlying illness often requires aggressive treatment: chemotherapy, bone marrow or peripheral blood stem cell transplantation. This could lead to severe and prolonged immunosuppression, increasing the risk of infection and possibly worsening the prognosis [1], [2]. Mortality attributable to hospital-acquired (HA) bacteraemia in patients with cancer ranges between 10 and 20% [3]–[5], whereas mortality from HA pneumonia is much higher, between 40 and 60% [6]–[8]. Over the past few decades, major progress has been made in the curative treatment of haematological malignancies which has resulted in reduced overall mortality [9]–[12], but also longer neutropenia and higher risk of infectious complications [13], [14].

HAI are important adverse events in the disease history of patients with haematological malignancies, sometimes culminating in death; they are also responsible for longer hospital stay and increased healthcare costs [15], [16]. However, few studies have examined the incidence of HAI in this adult patient population as most surveillance have focused only on bone marrow transplant patients or children [13], [17]–[25]. For example, Engelhart et al. noted an incidence of 25.3 HAI per 1000 patient-days at risk [20], and Urrea et al. reported 17.7 HAI per 1000 patient-days among paediatric haematology/oncology patients [21]. To the best of our knowledge, trends in HAI incidence in the general population of adult patients with haematological malignancies have been poorly investigated. The objectives of this prospective surveillance were: 1) to track trends in the incidence of HA pneumonia, bacteraemia and urinary tract infections (UTI) in patients with haematological malignancies; 2) to identify the common etiological pathogens for these infections.

## Methods

### Setting

Prospective surveillance of HA pneumonia, bacteraemia and UTI was implemented at the 42-bed haematology department of Edouard Herriot University Hospital in Lyon, France, between 1^st^ January 2004 and 31^st^ December 2010. This 1000-bed hospital is composed of 3 haematology adult acute care units. Each of these units can accommodate 14 patients in single rooms. In total, 16 rooms are equipped with laminar airflow (LAF), 14 rooms with positive pressure isolation and high-efficiency particulate air (HEPA) filtration, and 12 have no specific air system. Patients with acute myeloid leukaemia (AML) or acute lymphoid leukaemia (ALL) were selectively admitted in the department, whereas patients with lymphoma were more often admitted to another hospital.

### Data Sources

All patients hospitalized ≥48 hours in the haematology department between 1^st^ January 2004 and 31^st^ December 2010 were included in the surveillance. A standardized data sheet was filled for each patient, based on clinical observation and microbiological results. It recorded demographic and medical data: patient age, gender, dates of admission and discharge, diagnosis of the haematological disease, reason for admission. The main risk factors of infection were also recorded: neutropenia (<500 neutrophils/mm^3^) and its length, exposure to central venous catheterization (CVC), length of stay, hospitalization in non protected room. Surveillance was based on a local, standardized protocol derived from the French national protocol for HAI surveillance [26].

### HAI Diagnosis

The case definition of HA pneumonia was: 1) chest X-rays or chest computed tomography-scans exhibiting lung infiltrates; and 2) temperature >38°C or leukocyte count >12 000/mm^3^ or <4 000/mm^3^; and 3) at least 1 of the following: (a) sputum change, (b) suggestive auscultation, (c) low oxyhaemoglobin saturation, or (d) increased pulmonary oxygen consumption; and 4) confirmation of pneumonia diagnosis by: (a) the practitioner in charge of the patient; and/or (b) microbial identification of the potential causal microorganism. The etiological agents were isolated from sputum, or directed bronchoalveolar lavage, or protected specimen by telescopic brush or protected distal tracheal specimen by catheter, or histological sample [27].

The case definition of HA bacteraemia was: 1) at least 1 positive blood culture justified by clinical signs for most microorganisms; or 2) at least 2 positive blood cultures justified by clinical signs, at 2 different times, separated by less than 48 hours from each other, when the microorganism was 1 of the following: coagulase-negative *Staphylococci*, *Bacillus spp*. (except *Bacillus anthracis*), *Corynebacterium spp*., *Propionibacterium spp*., *Micrococcus spp*. [27].

The case definition of HA UTI was: 1) at least 1 of the following clinical signs/symptoms: fever (>38°C), urgency, increased urinary frequency, dysuria, burning on urination or pain in the lower abdomen, with no other recognized cause; and 2) a positive urine culture (≥10^5^ microorganisms/mm^3^) with 1 or 2 different pathogens isolated in case of urinary catheter exposure in the previous 7 days, or leukocyturia (≥10^4^ leukocytes/mm^3^) and positive urine culture (≥10^3^ microorganisms/mm^3^) with 1 or 2 different pathogens isolated if any urinary catheter was in place in the previous 7 days [27].

The causative microorganism for each infection was recorded. Only the first HAI per hospital stay that occurred ≥48 hours after patient admission was considered. Also, patient-days at risk were censored at first HAI, if it occurred.

### Statistical Analysis

Discrete variables were described as number and percentage, and continuous variables, as mean and standard deviation (SD). The HAI attack rate was the number of HAI per 100 patients, HAI incidence was the number of HAI per 1000 patients-days of hospitalization at risk. Poisson regression was fitted to assess temporal trends of HA pneumonia, bacteraemia and UTI incidence. The number of HAI was the dependent variable and the number of patient-days at risk was the offset. The independent variable of interest was the year of hospitalization. Potential confounders were: patient age, gender, neutropenia during hospital stay, CVC during hospital stay, diagnosis at admission, and main treatment received during hospitalization. Variables with *P*<0.15 after univariate analysis were entered in the initial multivariate model; then, a backward step-wise process was initiated, the models were compared with the likelihood ratio test. The significance level was *P*<0.05; all tests were 2-tailed. Statistical analysis was performed with Stata 10.0 (Stata Corp.). Data were recorded with EpiInfo, version 6.0. All data were analyzed anonymously.

## Results

### Patient Characteristics

From 2004 to 2010, 3 355 patients, accounting for 58 063 patient-days at risk, were included. The mean duration of hospital stay was 26 days (SD: 21.1 days). Overall, 1 873 (55.8%) were men and 1 482 (44.2%) were women (men/women gender ratio = 1.26). Mean age was 48.8 years (SD: 15.7 years). Patient characteristics are reported in Table 1. In total, 51.3% (n = 1704) had AML, 22.2% (n = 739) had ALL, and 9.5% (n = 315) had multiple myeloma. During their hospitalization, 66.7% of patients (n = 2 063) had neutropenia for a mean duration of 20.9 days (SD: 12.9 days). Most patients (89.7%, n = 2 574) had CVC and 56.9% (n = 1 909) were hospitalized in a protected room. In-hospital mortality was 5% (n = 159).

**Table 1:** Characteristics of patients hospitalized in the Haematology Department, Edouard Herriot Hospital, Lyon (France), 2004–2010.

Characteristics	Year							*P*	Overall (n = 3355)
	2004 (n = 617)	2005 (n = 547)	2006 (n = 546)	2007 (n = 437)	2008 (n = 393)	2009 (n = 413)	2010 (n = 402)		
**At admission**									
Gender, male	366 (59.3)	317 (58)	298 (54.6)	233 (53.3)	212 (53.9)	225 (54.5)	222 (55.2)	0.37	1,873 (55.8)
Age, years^a^	50.8 (15.9)	47.8 (16.1)	48.9 (15.8)	50.0 (15.4)	48.7 (15.6)	47.8 (14.9)	46.5 (15.6)	<0.001	48.8 (15.7)
Haematological disease								<0.001	
Acute myeloid leukaemia	299 (49.6)	252 (46.8)	260 (47.6)	249 (57.2)	230 (58.8)	208 (50.7)	206 (51.4)		1,704 (51.3)
Acute lymphoid leukaemia	118 (19.6)	124 (23.1)	93 (17)	64 (14.7)	97 (24.8)	128 (31.2)	115 (28.7)		739 (22.2)
Chronic lymphocytic leukaemia	13 (2.2)	8 (1.5)	6 (1.1)	11 (2.5)	6 (1.5)	7 (1.7)	2 (0.5)		53 (1.6)
Chronic myeloid leukaemia	28 (4.6)	11 (2)	14 (2.6)	8 (1.8)	5 (1.3)	4 (1)	5 (1.2)		75 (2.3)
Lymphoma	12 (2)	31 (5.8)	15 (2.8)	7 (1.6)	3 (0.8)	6 (1.5)	3 (0.7)		77 (2.3)
Myeloma	71 (11.8)	68 (12.6)	56 (10.3)	35 (8.1)	19 (4.9)	36 (8.8)	30 (7.5)		315 (9.5)
Myelodysplasia	19 (3.1)	15 (2.8)	22 (4)	18 (4.1)	4 (1)	8 (1.9)	4 (1.0)		90 (2.7)
Other	43 (7.1)	29 (5.4)	80 (14.7)	43 (9.9)	27 (6.9)	13 (3.2)	36 (9.0)		271 (8.1)
Reason for admission								<0.001	
Induction chemotherapy	139 (23.2)	102 (18.7)	117 (21.5)	112 (25.6)	81 (20.9)	71 (17.2)	85 (21.3)		707 (21.3)
Consolidation chemotherapy	146 (24.4)	147 (27.0)	119 (21.8)	111 (25.5)	119 (30.8)	148 (35.9)	146 (36.7)		936 (28.2)
Transplantation	86 (14.4)	87 (16.0)	65 (11.9)	70 (16)	63 (16.3)	79 (19.2)	72 (18.1)		522 (15.7)
Palliative	11 (1.8)	15 (2.7)	15 (2.75)	13 (3.0)	8 (2.1)	6 (1.5)	4 (1)		72 (2.2)
Infection suspicion	62 (10.4)	51 (9.4)	41 (7.5)	30 (6.9)	28 (7.2)	15 (3.6)	23 (5.8)		250 (7.5)
Graft rejection suspicion	9 (1.5)	11 (2)	9 (1.7)	10 (2.3)	8 (2.1)	9 (2.2)	7 (1.7)		63 (1.9)
Relapse suspicion	39 (6.5)	57 (10.4)	36 (6.6)	23 (5.3)	11 (2.8)	13 (3.1)	11 (2.8)		190 (5.7)
Other	106 (17.7)	75 (13.8)	143 (26.2)	67 (15.3)	69 (17.8)	71 (17.2)	50 (12.6)		581 (17.5)
**During hospitalization**									
Neutropenia (neutrophils <0.5G/L)	374 (66.9)	315 (59.4)	302 (58.3)	293 (70.1)	262 (71.8)	255 (72.2)	262 (74.9)	<0.001	2,063 (66.7)
Central venous catheterization	432 (88.9)	401 (80.2)	412 (87.1)	363 (87.9)	336 (96.6)	304 (96.5)	326 (97.6)	<0.001	2,574 (89.7)
Protected room^b^	241 (47.3)	246 (46.2)	313 (61.3)	282 (64.5)	276 (70.2)	273 (66.1)	278 (69.2)	<0.001	1,909 (56.9)
Length of stay, days^a^	23.3 (17.9)	23.2 (16.8)	24.6 (23.6)	29.3 (26.3)	28.8 (19.9)	27.2 (22.6)	28.3 (19.4)	<0.001	26.0 (21.1)
Length of neutropenia, days^a^	20.6 (14.4)	20.0 (12.2)	21.9 (14.3)	20.6 (11.9)	20.8 (11.9)	19.9 (12.9)	22.4 (12.0)	<0.001	20.9 (12.9)
Deceased	20 (3.7)	29 (5.5)	26 (4.9)	31 (7.4)	25 (6.6)	16 (4.1)	12 (3.3)	<0.001	159 (5.0)

Notes: Data are n (%), unless specified otherwise.

a Mean (SD).

b Protected room: laminar airflow or positive pressure isolation or high-efficiency particulate air filtration.

Between 2004 and 2010, variations in age (*P*<0.001), diagnosis of haematological disease (*P*<0.001), and reason for hospitalization (*P*<0.001) were observed but without linear trends. The proportions of patients with neutropenia or CVC exposure during hospital stay increased during the study period: from 66.9% of patients with neutropenia in 2004 to 74.9% in 2010 (*P*<0.001), and from 88.9% of patients with CVC in 2004 to 97.6% in 2010 (*P*<0.001).

### HAI Incidence

**Figure 1:**
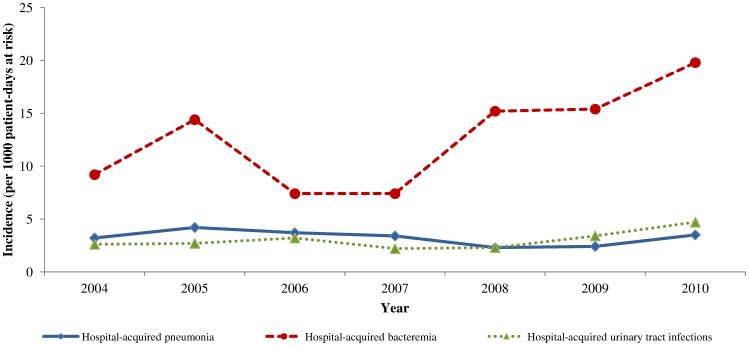
Trends in the incidence of hospital-acquired infections, Haematology Department, Edouard Herriot Hospital, Lyon (France) 2004–2010.

In total, 1 055 (31.4%) patients had HAI: 191 (5.7%) had pneumonia, 694 (20.7%) had bacteraemia and 170 (5.1%) had UTI. Table 2 describes the incidence by site of infection, year and haematological disease. Figure 1 depicts trends of incidence over the years. The overall HAI attack rate was 31.4 per 100 patients and its incidence was 18.2 per 1000 patient-days at risk (95% confidence interval (95% CI): 17.1–19.3). The HA pneumonia attack rate was 5.7 per 100 patients and its incidence was 3.3 per 1000 patient-days at risk (95% CI: 2.8–3.8). The HA bacteraemia attack rate was 20.7 per 100 patients and its incidence was 12.0 per 1000 patient-days at risk (95% CI: 11.1–12.9). The HA UTI attack rate was 5.1 per 100 patients and its incidence was 2.9 per 1000 patient-days at risk (95% CI: 2.5–3.4). HA pneumonia incidence was higher among AML patients (incidence: 4.2‰ patient-days, 95% CI 3.5–4.9) compared to ALL patients (incidence: 1.8‰ patient-days, 95% CI 1.1–2.7). HA bacteremia incidence and UTI incidence were similar according to patient haematological diagnosis.

**Table 2:** Annual incidence of hospital-acquired infections, Haematology Department, Edouard Herriot Hospital, Lyon (France) 2004–2010.

Characteristics	2004	2005	2006	2007	2008	2009	2010	Overall
**Pneumonia**, n (attack rate^a^)								
Overall population	33 (5.4)	35 (6.4)	36 (6.6)	32 (7.3)	17 (4.3)	16 (3.9)	22 (5.5)	191 (5.7)
AML patients	28 (9.4)	27 (10.7)	24 (9.2)	24 (9.6)	13 (5.7)	9 (4.3)	14 (6.8)	139 (8.2)
ALL patients	0 (0)	3 (2.4)	8 (8.6)	2 (3.1)	1 (1.0)	2 (1.6)	3 (2.6)	19 (2.6)
Other patients	5 (2.7)	5 (3.1)	4 (2.1)	6 (4.9)	3 (4.7)	5 (6.8)	5 (6.3)	33 (3.7)
Pneumonia incidence rate^b^ (95% CI)								
Overall	3.2 (2.2–4.5)	4.2 (3.0–5.8)	3.7 (2.7–5.1)	3.4 (2.4–4.7)	2.3 (1.4–3.6)	2.4 (1.4–3.8)	3.5 (2.3–5.3)	3.3 (2.8–3.8)
AML patients	4.9 (3.3–6.9)	6.0 (4.1–8.7)	4.2 (2.8–6.2)	4.0 (2.6–5.9)	3.0 (1.7–5.0)	2.3 (1.1–4.3)	4.1 (2.3–6.7)	4.2 (3.5–4.9)
ALL patients	0 (–)	2.0 (0.5–5.5)	5.2 (2.4–9.9)	1.8 (0.3–5.8)	0.6 (0.0–2.8)	1.2 (0.2–3.9)	2.0 (0.5–5.4)	1.8 (1.1–2.7)
Other patients	1.6 (0.6–3.6)	2.4 (0.8–4.7)	1.6 (0.5–3.9)	2.5 (1.0–5.3)	2.5 (0.6–6.7)	4.0 (1.5–8.9)	3.8 (1.4–8.5)	2.4 (1.6–3.3)
**Bacteremia**, n (attack rate^a^)								
Overall population	95 (15.4)	119 (21.8)	71 (13.0)	70 (16.0)	112 (28.5)	104 (25.2)	123 (30.6)	694 (20.7)
AML patients	61 (20.4)	70 (27.8)	44 (16.9)	45 (18.1)	75 (32.6)	60 (28.8)	75 (36.4)	430 (25.2)
ALL patients	17 (14.4)	26 (21.0)	16 (17.2)	13 (20.3)	25 (25.8)	20 (15.6)	25 (21.7)	142 (19.2)
Other patients	17 (9.1)	23 (14.2)	11 (5.7)	12 (9.8)	12 (18.8)	24 (32.4)	23 (28.8)	122 (13.8)
Bacteremia incidence rate^b^ (95% CI)								
Overall	9.2	14.4	7.4	7.4	15.2	15.4	19.8	12.0
	(7.5–11.2)	(11.9–17.1)	(5.8–9.2)	(5.8–9.3)	(12.6–18.2)	(12.6–18.5)	(16.5–23.5)	(11.1–12.9)
AML patients	10.6	15.7	7.8	7.6	17.2	15.6	22.1	12.9
	(8.2–13.5)	(12.3–19.7)	(5.7–10.4)	(5.6–10.0)	(13.6–21.4)	(12.0–20.0)	(17.5–27.5)	(11.7–14.1)
ALL patients	11.6	17.6	10.4	11.4	13.9	11.8	16.5	13.4
	(7.0–18.1)	(11.8–25.5)	(6.2–16.5)	(6.3–19.0)	(9.2–20.3)	(7.4–18.0)	(10.9–24.0)	(11.3–15.7)
Other patients	5.6 (3.4–8.7)	9.8 (6.4–14.5)	4.5 (2.4–7.8)	5.0 (2.7–8.6)	9.8 (5.3–16.8)	19.4(12.7–28.4)	17.6(11.4–26.0)	8.7 (7.3–10.4)
**Urinary tract infection**, n (attack rate^a^)								
Overall population	27 (4.4)	22 (4.0)	31 (5.7)	21 (4.8)	17 (4.3)	23 (5.6)	29 (7.2)	170 (5.1)
AML patients	11 (3.7)	11 (4.4)	18 (6.9)	11 (4.4)	11 (4.8)	11 (5.3)	14 (6.8)	87 (5.1)
ALL patients	8 (6.8)	6 (4.8)	5 (5.4)	5 (7.8)	1 (1.0)	5 (3.9)	7 (6.1)	37 (5.0)
Other patients	8 (4.3)	5 (3.1)	8 (4.1)	5 (4.1)	5 (7.8)	7 (9.5)	8 (10.0)	46 (5.2)
Urinary infection incidence rate^b^ (95% CI)								
Overall	2.6 (1.8–3.8)	2.7 (1.7–4.0)	3.2 (2.2–4.5)	2.2 (1.4–3.3)	2.3 (1.4–3.6)	3.4 (2.2–5.0)	4.7 (3.2–6.6)	2.9 (2.5–3.4)
AML patients	1.9 (1.0–3.3)	2.5 (1.3–4.3)	3.2 (1.9–4.9)	1.8 (1.0–3.2)	2.5 (1.3–4.4)	2.9 (1.5–5.0)	4.1 (2.3–6.7)	2.6 (2.1–3.2)
ALL patients	5.4 (2.5–10.3)	4.1 (1.7–8.5)	3.3 (1.2–7.2)	4.4 (1.6–9.7)	0.6 (0.0–2.8)	3.0 (1.1–6.6)	4.6 (2.0–9.1)	3.5 (2.5–4.8)
Other patients	2.6 (1.2–5.0)	2.1 (0.8–4.7)	3.3 (1.5–6.2)	2.1 (0.8–4.7)	4.1 (1.5–9.1)	5.7 (2.5–11.2)	6.1 (2.8–11.6)	3.3 (2.4–4.4)

Notes: AML, acute myeloid leukaemia; ALL, acute lymphoid leukaemia; 95%CI, 95% confidence interval.

a number of diagnosed infection/100 patients;

b number of diagnosed infection/1000 patient-days at risk.

Univariate Poisson regression indicated that the incidence of HA pneumonia was stable between 2004 and 2010: −4% per year (95% CI: −11%, +3%, *P* = 0.23). HA bacteraemia increased by 12% per year (95% CI: +8%, +16%, *P*<0.001). A trend for increased HA UTI was observed: +7% per year (95% CI: 0%, +16%, *P* = 0.08).

Multivariate Poisson regression models after controlling for neutropenia and medical treatment showed a stable incidence rate for HA pneumonia over years: +5% per year (95% CI: −12%, +2%, *P* = 0.17). Multivariate Poisson regression models after controlling for neutropenia, CVC and diagnosis of haematological disease showed an increase by 11% per year (95% CI: +6%, +15%, *P*<0.001). Multivariate Poisson regression models after controlling for patient gender showed a stable incidence rate for HA UTI: +6% per year (95% CI: −1%, +15%, *P* = 0.11).

### Microbiology

**Figure 2:**
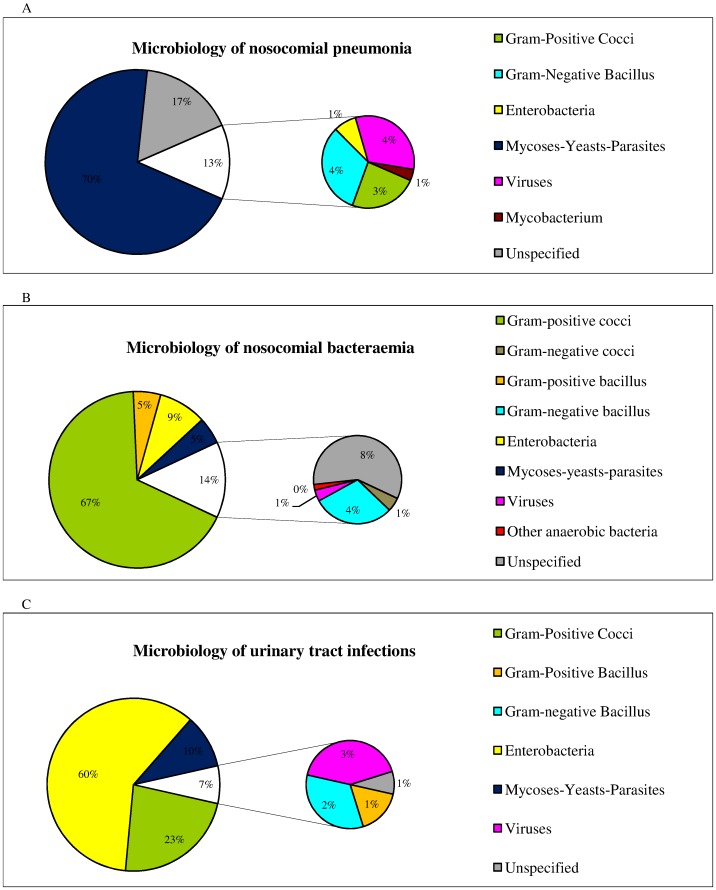
A. Distribution of the isolated pathogens in hospital-acquired pneumonia, Haematology Department, Edouard Herriot Hospital, Lyon (France) 2004–2010. B. Distribution of the isolated pathogens in hospital-acquired bacteraemia, Haematology Department, Edouard Herriot Hospital, Lyon (France) 2004–2010. C. Distribution of the isolated pathogens in hospital-acquired urinary tract infections, Haematology Department, Edouard Herriot Hospital, Lyon (France) 2004–2010.

Figure 2 reports the distribution of microorganisms by infection site. The causative pathogens isolated most frequently in HA pneumonia were *Aspergillus spp.* (59.2%, n = 113) and *Candida* (7.8%, n = 15). Viral HA pneumonia affected 8 patients overall (4.2%). The causative pathogens isolated most frequently in HA bacteraemia were coagulase-negative *Staphylococcus* (44.2%, n = 307) and *Candida* (4.6%, n = 32). Hospital-acquired blood infection by a virus concerned 7 patients (1.0%). The causative pathogens isolated most frequently in HA UTI were enterobacteria (60%, n = 102). Viral HA UTI affected 5 patients (2.9%).

## Discussion

The study’s objective was to describe trends in HA pneumonia, bacteraemia and UTI incidence between 2004 and 2010 as they were the most frequent and lethal sites of infection in patients with haematological malignancies [17], [20], [22], [28]. No significant trend was found in HA pneumonia and UTI incidence. HA bacteraemia incidence increased by 11% per year independently of neutropenia, CVC and patient’s haematological disease. Table 3 compares our results with 3 previous surveillance studies about neutropenic cancer patients [13] and patients with haematological malignancies [19], [20].

**Table 3:** Comparison with other studies.

	Carlisle et al. 1993 [13]	Dettenkofer et al.2003 [19]	Engelhart et al.2002 [20]	Present study
Number of patients	920 neutropenic patients	351 patients	116 patients	3,355 patients
Study period	42 months	54 months	8 months	84 months
Pneumonia	5.5 per 100 neutropenic patients	22 per 100 patients	12.9 per 100 patients	5.7 per 100 patients
Incidence rate	–	5.4 per 1000 patient-days	9.7 per 1000 patient-days at risk	3.3 per 1000 patient-days at risk
Bacteraemia	13.5 per 100 neutropenic patients	24 per 100 patients	16.4 per 100 patients	20.7 per 100 patients
Incidence rate	–	6.0 per 1000 patient-days	11.2 per 1000 patient-days at risk	12.0 per 1000 patient-days at risk
Urinary tract infections	5.7 per 100 neutropenic patients	3.0 per 100 patients	6.0 per 100 patients	5.1 per 100 patients
Incidence rate	–	0.8 per 1000 patient-days	3.0 per 1000 patient-days at risk	2.9 per 1000 patient-days at risk

Engelhart et al. reported data on HAI and fever of unknown origin among adult haematology and oncology patients [20]. They discerned a higher pneumonia incidence (9.7 per 1000 patient-days), while most rooms in their department were not equipped with LAF or positive pressure isolation and HEPA filtration. Consequently, protection against *Aspergillus*, the most common pathogen in HA pneumonia in this patient group, would be less efficient [29], [30]. In our study, the globally-stable incidence of HA pneumonia could be balanced by decreased invasive aspergillosis during the same period [31]. Consequently, it is not clear whether pneumonia of other origins was truly stable. However, we found a high rate of *Aspergillus* pneumonia (59.2% of the causative agents in pneumonia) although 56.9% of the patients were hospitalized in a protected room. *Aspergillosis* remained a large concern among our patients and *Aspergillus* pneumonia occurred mainly in immunosuppressed patients like AML or ALL patients [31], [32]. Moreover, in our survey, incidence of HA-pneumonia appeared higher among AML patient compared with ALL patients or other patients.

The incidence of bacteraemia rose during the study period. Bacteraemia are mostly of endogenous origin, caused by pathogens or saprophytic cutaneous flora, such as *Staphyloccocus spp.* [33]. Over the years, we noted a higher proportion of patients with CVC, well-known to be a major risk factor for HA bloodstream infection [23]. The length and depth of the neutropenic phase, especially during chemotherapy, could also be related to incidence of bacteraemia. Nevertheless, the increased HA bacteraemia incidence was independent of CVC-exposure or neutropenia. Other factors could explain this increase, like more intense chemotherapy over the years, changes in antibiotic prophylaxis or hygiene practices. However, a previous study reported a protective impact of hygiene measures [34]. Then, the increase of incidence might be mostly related to the severity of patients or to their underlying diseases. Carlisle et al. recorded a lower HA bacteraemia rate than what we observed in our department [13]. They implemented 42-month surveillance and detected 13.5 bacteraemia per 100 neutropenic days. However, their study focused on neutropenic cancer patients and not on a specific population hospitalized with haematologic malignancies, the last population could have more severe immune system dysfunction related to their disease and not only chemotherapy. On the other hand, we found a similar incidence of bacteraemia as Engelhart et al. (11.2 per 1000 patient-days at risk) who investigated patients with haematological-oncologic diseases [20].

UTI incidence was stable. We encountered a similar incidence as Carlisle et al. (5.7 per 100 neutropenic patients) [13] and Engelhart et al. (6.0 per 100 patients) [20]. Dettenkofer et al. reported a much lower UTI rate (3.0 per 100 patients, 0.8 per 1,000 patient-days) in transplant patients [19], but all of them received antimicrobial prophylaxis, which could have reduced the risk of infection in prolonged neutropenia cases [35]–[37].

Our study had some limitations. First, the study population was mainly composed of AML and ALL patients. Our findings cannot be generalized to all haematological populations. Secondly, no post-discharge surveillance was undertaken, which could have led to underestimation of the incidence because infections with long incubation periods could have been missed. However, it did not affect trends because possible underestimation could be constant over the years. Furthermore, some potential risk factors for infection were not collected in our surveillance database, like length of CVC exposure, mechanical ventilation, type of stem-cell transplant (autologous or allogenous).

Its main strength was prospective data collection, with standardized case definitions. Ours was a single-centre study, which permitted us to compare trends and reinforced internal validity. Moreover, the true incidence was assessed because all patients were followed until their discharge. Few investigations have analyzed data over such a long time period and in such a large population, which increased the study’s power calculation. Finally, major confounding factors were taken into account with multivariate analysis.

In summary, we observed that the incidence of HA pneumonia and HA UTI remained stable. This could be explained by improvement of infection control measures, and took account of patient exposure to more aggressive chemotherapies. The incidence of bacteraemia increased independently of CVC exposure or neutropenia, which are known risk factors of bacteraemia. Further etiological studies are needed to explore in depth the factors that could explain this trend.
